# Rescue nasopharyngeal tube for preterm infants non-responsive to initial ventilation after birth

**DOI:** 10.1038/s41390-024-03033-6

**Published:** 2024-01-25

**Authors:** Carina Belting, Christoph M. Rüegger, Andreas D. Waldmann, Dirk Bassler, Vincent D. Gaertner

**Affiliations:** 1 Newborn Research, Department of Neonatology, University Hospital and University of Zürich, Zürich, Switzerland; 2 Department of Pediatric Intensive Care and Neonatology, Children’s Hospital of Eastern Switzerland, St. Gallen, Switzerland; 3 Department of Anaesthesiology and Intensive Care Medicine, Rostock University Medical Centre, Rostock, Germany; 4 Division of Neonatology, Dr von Hauner Children’s Hospital, Ludwig-Maximilians-Universität München, Munich, Germany

## Abstract

**Background:**

Physiological changes during the insertion of a rescue nasopharyngeal tube (NPT) after birth are unclear.

**Methods:**

Observational study of very preterm infants in the delivery room. Data were extracted at predefined timepoints starting with first facemask placement after birth until 5 min after insertion of NPT. End-expiratory lung impedance (EELI), heart rate (HR) and SpO_2_/FiO_2_-ratio were analysed over time. Changes during the same time span of NIPPV via facemask and NIPPV via NPT were compared.

**Results:**

Overall, 1154 inflations in 15 infants were analysed. After NPT insertion, EELI increased significantly [0.33 AU/kg (0.19–0.57), *p* < 0.001]. Compared with the mask period, changes in EELI were not significantly larger during the NPT period [median difference (IQR) = 0.14 AU/kg (−0.14–0.53); *p* = 0.12]. Insertion of the NPT was associated with significant improvement in HR [52 (33–96); *p* = 0.001] and SpO_2_/FiO_2_-ratio [161 (69–169); *p* < 0.001] not observed during the mask period.

**Conclusions:**

In very preterm infants non-responsive to initial facemask ventilation after birth, insertion of an NPT resulted in a considerable increase in EELI. This additional gain in lung volume was associated with an immediate improvement in clinical parameters. The use of a NPT may prevent intubation in selected non-responsive infants.

**Impact:**

- After birth, a nasopharyngeal tube may be considered as a rescue airway in newborn infants non-responsive to initial positive pressure ventilation via facemask. Although it is widely used among clinicians, its effect on lung volumes and physiological parameters remains unclear.
- Insertion of a rescue NPT resulted in a considerable increase in lung volume but this was not significantly larger than during facemask ventilation. However, insertion of a rescue NPT was associated with a significant and clinically important improvement in heart rate and oxygenation.
- This study highlights the importance of individual strategies in preterm resuscitation and introduces the NPT as a valid option.

## Introduction

In apnoeic or bradycardic infants in the delivery room, the use of non-invasive positive pressure ventilation (NIPPV) is recommended.^1^ In case of ineffective ventilation, airway management should be optimised, including consideration of an alternative airway.^2,3^

Alternative airways, including the nasopharyngeal tube (NPT) have gained popularity over the past years, mainly to avoid adverse effects of intubation and subsequent mechanical ventilation but potentially also because of a decreased proficiency in endotracheal intubation.^4–8^ If used as primary interface after birth, the NPT yielded similar intubation rates and similar morbidity and mortality, but larger leak, more airway obstructions and inadequate tidal volumes, when compared with a facemask.^9–11^ However, the included trials investigated the NPT as the primary interface after birth, and its effect as rescue airway to prevent impending intubation in non-responsive preterm infants is unclear.

Electrical impedance tomography (EIT) is a radiation-free bedside tool measuring changes in global and regional lung volumes in a breath-by-breath analysis by using the different electrical properties of air and fluid.^12^ EIT measurements of lung volumes at nipple level has been shown to be representative for the whole lung in ventilated preterm infants,^13^ and changes in end-expiratory lung impedance (ΔEELI) correspond to changes in functional residual capacity (ΔFRC).^14^

In this study, we used data from a recent randomised controlled trial comparing surfactant nebulization (SN) with standard care to assess the development of lung volumes and cardiorespiratory parameters before, during and after the introduction of an NPT in infants who are non-responsive to initial facemask ventilation after birth. Specifically, we aimed to determine whether recruitment of FRC would be more effective with an NPT than with a facemask. Finally, we assessed differences between infants who received SN vs standard care.

## Methods

This is a secondary analysis of a previously published randomised controlled trial conducted at the University Hospital Zurich, comparing the effect of prophylactic surfactant nebulisation with standard care in preterm infants between 26 0/7 and 31 6/7 weeks’ gestation.^15^ The trial and this secondary analysis were approved by the Cantonal Ethics Committee Zurich (KEK-2020-00890). Antenatal written informed consent was obtained from all parents.

### Population and intervention

The setup of the original study has been described previously.^15^ After delayed cord clamping for 60 s, infants were supported on continuous positive airway pressure support with a distending pressure of 8 mbar [6 mmHg] using the EVE NEO ventilator (Fritz Stephan GmbH, Gackenbach, Germany) and a facemask (ComfortStar, Dräger Medical System, Lübeck, Germany). It was the clinician’s decision to increase pressure levels, apply NIPPV or change the interface to an appropriately sized NPT (Vygon, Ecouen, France) depending on their evaluation of the infant’s clinical appearance. Infants randomised to the intervention group received 200 mg/kg surfactant via a nebuliser positioned between the interface and the ventilator starting with the first application of a facemask. Infants randomised to the control group received positive distending pressure only. Infants who required intratracheal surfactant (via endotracheal tube or thin catheter) within the first 30 min after birth were excluded from the original study. For the current secondary analysis, data of infants who were non-responsive to initial facemask ventilation and who received NIPPV via NPT were used, irrespective of their allocated group assignment.

### Data collection

As soon as the newborn reached the resuscitaire, a textile EIT belt was fastened around the thorax at nipple level. EIT data were recorded at a frame rate of 51 Hz using the LuMon device (SenTec AG, Landquart, Switzerland).^14,16^ During resuscitation, the infant’s body and the operator’s hands were video recorded from above. A flow sensor with an accuracy of 5% was placed between the T-piece device and the facemask to continuously measure airway pressure and flow. Fraction of inspired oxygen (FiO_2_) was measured by an oxygen analyser (AX300, Teledyne Analytical Instruments, California) in the inspiratory limb of the ventilator. Heart rate (HR) and preductal peripheral oxygen saturation (SpO_2_) were recorded using a Masimo Radical 7 pulse oximeter set to a 2-s averaging time and maximum sensitivity (Masimo Corporation, Irvine, California). Respiratory function parameters were recorded at 200 Hz using the NewLifeBox recording system (Advanced Life Diagnostics, Weener, Germany).

### Data analysis

Video recordings were used to detect interface changes during primary stabilisation. Using airway flow data in Pulmochart software (Advanced Life Diagnostics, Weener, Germany), the exact beginning of each NIPPV sequence was identified.

Clinical, physiological and EIT data were extracted over a timeframe of 20 s for the following six predefined events (illustrated in Fig. 2a): first, initial application of a facemask (*mask on*, baseline); second, start of NIPPV (*start NIPPV mask* – in some patients equal to *mask on*); third, removal of facemask before NPT insertion (*mask off*). The time span between *start NIPPV mask* and *mask off* was labelled *mask period* and the same time span was selected during NPT ventilation (*NPT period*). The fourth timepoint is the insertion of the NPT (*NPT in*) and the fifth timepoint is an individual follow-up timepoint (*NPT FU*), received by adding the duration of *mask period* to *NPT in* (the difference between fourth and fifth timepoint corresponds to *NPT period*). This was done individually for each patient in order to compare changes during the same time spans. Finally, a sixth timepoint included follow-up data 5 min after insertion of the NPT (*NPT 5* *min*). Changes over time and differences between corresponding timespans (i.e., *mask period* and *NPT period*) were assessed. We chose the comparison of the two timespans to account for the time effect on our results. If an event took place within the timeframe of a previous event, data were included in both events. Data of infants who required intubation were only used until this point.

EIT data during artefact-free tidal ventilation were extracted and analysed using ibex (version 1.4, SenTec AG, Landquart, Switzerland), including the following steps: First, EIT signals outside of predefined anatomical lung regions based on the vendor-provided human model chest atlas were excluded.^17–19^ Second, artefact-free breaths were manually identified in the EIT raw signal and included in further analysis. Third, EIT signals were normalised for body weight and calculated in arbitrary units per kilogram (AU/kg).^20^ Fourth, for each defined event, EELI was compared to the patient’s individual baseline (=*mask on*). Finally, changes in EELI were calculated for the *mask period* (*mask off* minus *start NIPPV mask*) and the equally long *NPT period* (*NPT FU* minus *NPT in*).

Pulmonary waveforms were analysed breath-by-breath. Spontaneous breaths were analysed before the beginning of NIPPV and subsequently, only NIPPV inflations were analysed. Breaths with negative V_T_ generated by concurrent spontaneous breaths were not considered for analysis.^21^ Time points and time spans corresponded to EIT data analysis.

### Statistical analysis

Data analysis was performed using R statistics (version 4.2.1).^22^ Parametric data are presented as mean and standard deviation and non-parametric data are presented as median and interquartile range (IQR). Differences in medians of EELI during *mask period* vs *NPT period* were analysed using the paired Wilcoxon test. Changes in physiological data over time were assessed using Friedman’s test. In case of a significant global difference, post-hoc analyses were performed and corrected for multiple comparisons using the Bonferroni-Holm method. For comparison of the intervention and control group, a median/quantile regression analysis with cluster-corrected standard errors was used to account for within subjects variance using the *rq*-package in R statistics. *P* < 0.05 were considered statistically significant.

## Results

### Population

**Fig. 1:**
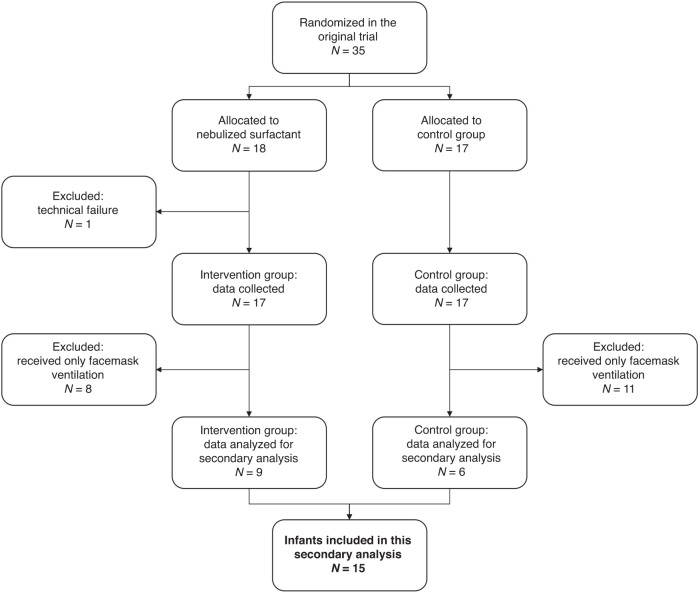
Flow chart of included patients.

Overall, 15 of 35 patients randomised in the original trial (43%) received NIPPV via NPT in the delivery room and were included in the current analysis (Fig. 1). Data for single events were excluded for various reasons (intubation before event, technical problems, flow sensor not connected/calibrated), leaving 89, 75, and 74 events for the analysis of EIT, physiological and respiratory function data, respectively. Overall, 1154 inflations were analysed for flow parameters and due to artefact exclusion, EIT data were restricted to 521 breaths. Patient characteristics are shown in Table 1.

**Table 1:** Baseline demographics and patient characteristics (*N* = 15).

Patient characteristics	Population (*N* = 15)
*Demographic*	
Gestational age at birth [weeks]	28.9 (26.9–29.4)
Birth weight [g]	950 (820–981)
Male, *n* (%)	3 (20)
*Prenatal*	
Completed antenatal steroids, *n* (%)	11 (73)
PPROM, *n* (%)	6 (40)
Chorioamnionitis, *n* (%)	4 (27)
Oligo- or anhydramnios, *n* (%)	5 (33)
Preeclampsia, *n* (%)	5 (33)
IUGR, *n* (%)	4 (27)
Delivery by CS, *n* (%)	14 (93)
*Postnatal*	
Apgar score at 5 min	5 (4–8)
Umbilical artery pH	7.33 (7.31–7.37)
Nebulised surfactant in the delivery room, *n* (%)	9 (60)
NIPPV as primary respiratory suport, *n* (%)	5 (33)
*Short-term respiratory outcomes*	
Intubation in the delivery room, *n* (%)	3 (20)^a^
Intubation within 24 h, *n* (%)	4 (27)
Intubation within 72 h, *n* (%)	6 (40)
Intubation during hospitalisation, *n* (%)	6 (40)
*Safety*	
Airleak, *n* (%)	2 (13)

Unless otherwise specified, median and interquartile range (IQR) are depicted.

*PPROM* prolonged premature rupture of the membranes; *IUGR* intrauterine growth restriction; *CS* caesarean section; *NIPPV* non-invasive positive pressure ventilation.

^a^One patient was intubated before event 6, and data for this event was excluded from the analysis. Two patients were intubated after event 6.

### Timing of facemask placement and insertion of NPT

The median (IQR) time to first facemask placement was 90 (78–119) s, duration of NIPPV via facemask was 147 (106–218) s and the time at insertion of the NPT was 342 (275–387) s. In five apnoeic patients (33%), NIPPV was initiated at first facemask placement.

### Lung volume changes over time

**Fig. 2:**
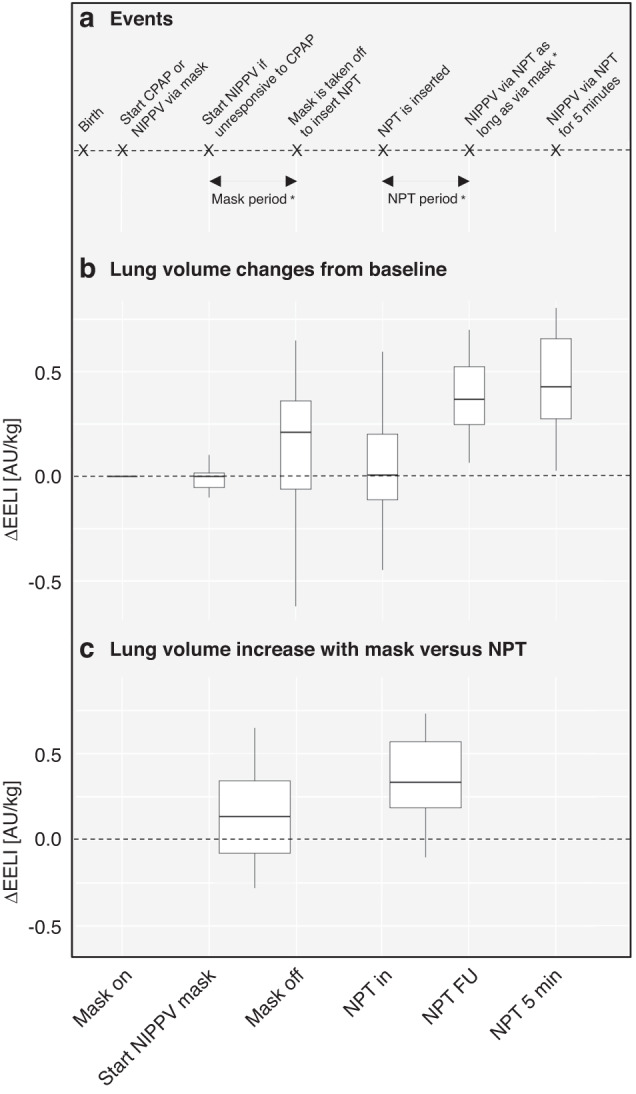
Changes in end-expiratory lung impedance over time. **a** Explanation of selected timepoints and time periods. **b** Changes in end-expiratory lung impedance are depicted at six predefined time-points compared to each patients individual baseline. **c** Changes in EELI over the same duration with each of the two interfaces. * = the length of *mask period* and *NPT period* is of individual length in each patient. Abbreviations: CPAP continuous positive airway pressure, NIPPV non-invasive positive pressure ventilation, NPT nasopharyngeal tube, ΔEELI change in end-expiratory lung impedance compared to baseline, AU/kg arbitrary units per kilogram, NPT FU individual follow-up timepoint (received by adding the duration of *mask period* to *NPT in*).

Over the six predefined timepoints, ΔEELI changed significantly (Friedman’s test, *p* < 0.001, Fig. 2b, Table 2). This was mainly attributable to the insertion of the NPT as ΔEELI was higher at *NPT FU* and *NPT 5* *min* compared to all other timepoints (both *p* < 0.001, Table 2).

**Table 2:** Changes of EELI compared to baseline and to previous event.

	Changes to baseline		Changes to previous event	
	ΔEELI [AU/kg] median (IQR) difference	*P*_adj_	ΔEELI [AU/kg] median (IQR) difference	*P*_adj_
baseline	-	-	-	-
start NIPPV mask	0.0 (−0.05 to 0.02)	0.916	0.0 (−0.05 to 0.02)	0.916
mask off	0.21 (−0.07 to 0.36)	0.447	0.14 (−0.08 to 0.34)	0.447
NPT in	0.01 (−0.11 to 0.20)	1.0	−0.10 (−0.20 to 0.02)	0.225
NPT FU	0.37 (0.25 to 0.52)	**<0.001**	0.33 (0.19 to 0.57)	**<0.001**
NPT 5 min	0.42 (0.28 to 0.66)	**<0.001**	0.05 (−0.09 to 0.21)	0.447

Bold﻿ indicates significant changes.

With the two different interfaces, median (IQR) increase in ΔEELI was 0.14 AU/kg (−0.08 to 0.34, *p* = 0.252) during *mask period* and 0.33 AU/kg (0.19 to 0.57, *p* < 0.001) during *NPT period* (median difference 0.14 AU/kg (−0.14 to 0.53, *p* = 0.1205, see Fig. 2c, Table 3)). Changes in EELI for the individual patients are provided in Supplementary Fig. S1. There was no difference in ∆EELI between intervention and control group over the course of NPT insertion [*t* = 0.55 *p* = 0.58].

**Table 3:** Changes of physiological and respiratory parameters with respective interface and differences between the two timespans.

	Mask period		NPT period		Difference	
	Median (IQR)	*P* value	Median (IQR)	*P* value	Median (IQR)	*P* value
ΔEELI [AU/kg]	0.14 (−0.08 to 0.34)	0.252	0.33 (0.19 to 0.57)	**<0.001**	0.14 (−0.14 to 0.53)	0.121
ΔSpO_2_/FiO_2_	−106 (−214 to −41)	**<0.001**	161 (69 to 169)	**<0.001**	233 (150 to 361)	**<0.001**
ΔHeart rate (bpm)	−2 (−12 to 19)	0.831	52 (33 to 96)	**0.001**	31 (−1 to 86)	0.064
ΔPEEP (cmH_2_O)	−0.4 (−0.6 to −0.1)	**0.01**	0.4 (0.0 to 0.7)	0.09	0.9 (0.5 to 1.3)	**0.021**
ΔPIP (IQRcmH_2_O)	8.5 (5.9 to 11.6)	**<0.001**	−0.4 (−5.9 to 0.3)	0.167	−11.6 (−13.6 to −6.6)	**<0.001**
ΔMAP (cmH_2_O)	2.3 (1.4 to 2.7)	**<0.001**	0.01 (−1.4 to 0.04)	0.340	−2.8 (−3.5 to −1.3)	**<0.001**
ΔV_T_ (ml/kg)	0.1 (−0.1 to 2.2)	0.326	−1.9 (−3.2 to 0.2)	0.190	−3.5 (−3.7 to 0.1)	0.094

The first and second columns show changes of EELI from baseline during NIPPV via facemask and NIPPV via NPT. The third column shows the difference between the two periods. Bold﻿ indicates significant changes.

### Changes in clinical and physiological parameters

**Fig. 3:**
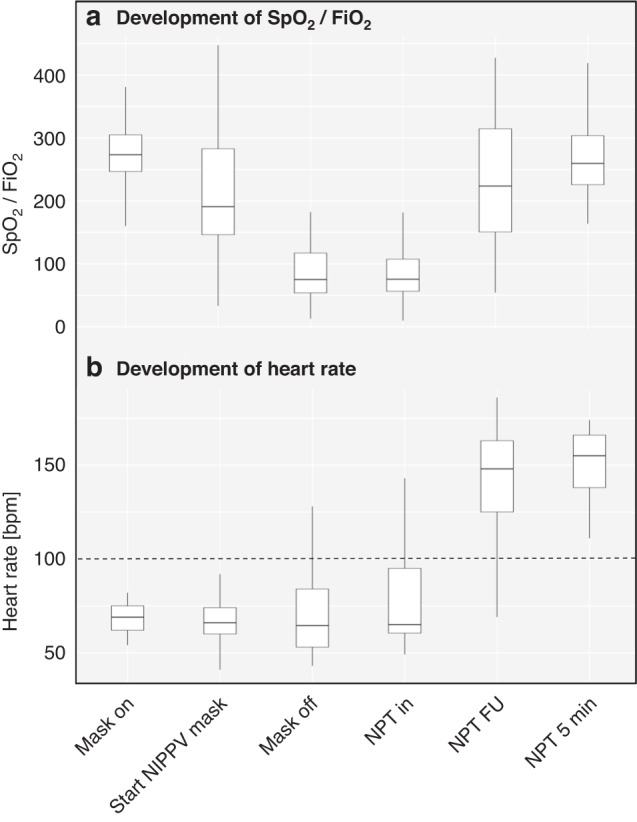
Development of SpO_2_/FiO_2_-ratio (a) and heart rate (b) over time. Abbreviations: SpO_*2*_ peripheral oxygen saturation, FiO_*2*_ fraction of inspired oxygen, bpm beats per minute. Explanation of time points (x-axis): mask on = start CPAP or NIPPV via mask, start NIPPV mask = start NIPPV if unresponsive to CPAP, mask off = mask is taken off to insert NPT, NPT in = NPT is inserted, NPT FU = NIPPV via NPT as long as via mask, NPT 5 min = NIPPV via NPT for 5 min.

Both, SpO_2_/FiO_2_ ratio (Friedman’s test, *p* < 0.001) and HR (Friedman’s test, *p* = 0.002) changed significantly over time, mostly attributable to the insertion ot the NPT. During *NPT period* SpO_2_/FiO_2_ ratio increased by 161 (69–169, *p* < 0.001) and HR increased by 52 bpm (33–96, *p* = 0.001; Fig. 3, Table 3). Eight of nine (89%) bradycardic infants became normocardic after introduction of the NPT (supplementary fig. S2).

**Fig. 4:**
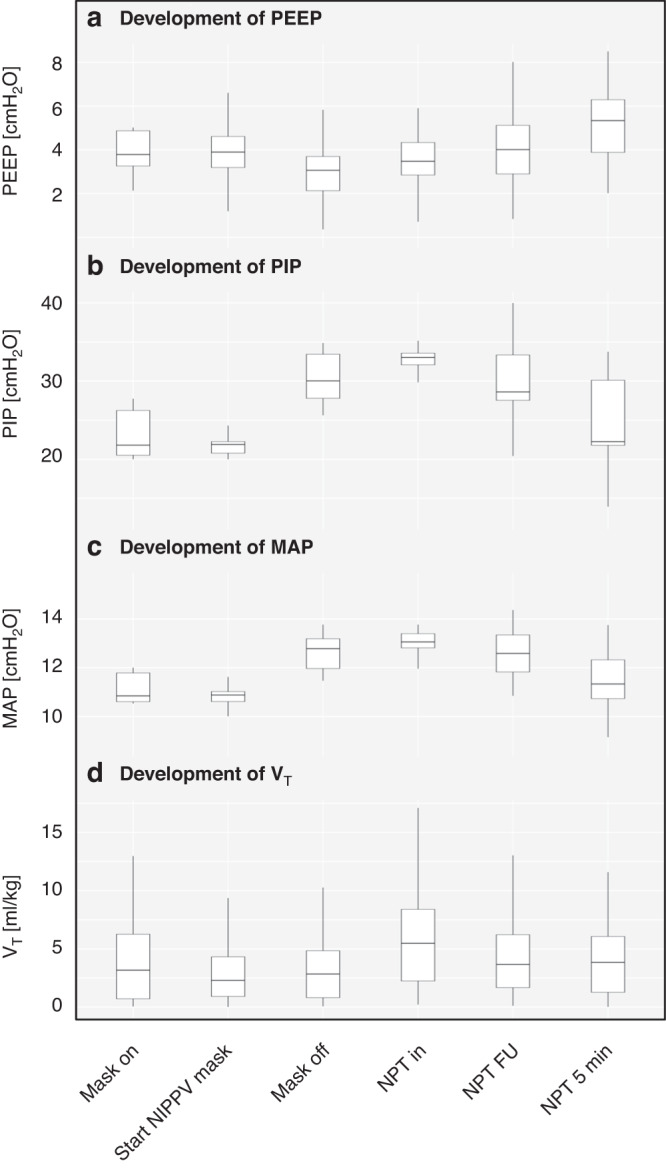
Development of respiratory parameters over time. This figure illustrates the development of PEEP (**a**), PIP (**b**), MAP (**c**) and V_T_ (**d**). Abbreviations: PEEP positive end-expiratory pressure, PIP positive inspiratory pressure, MAP mean airway pressure, V_T_ measured exhaled tidal volume. Explanation of time points (x-axis): mask on = start CPAP or NIPPV via mask, start NIPPV mask = start NIPPV if unresponsive to CPAP, mask off = mask is taken off to insert NPT, NPT in = NPT is inserted, NPT FU = NIPPV via NPT as long as via mask, NPT 5 min = NIPPV via NPT for 5 min.

Airway pressures changed significantly over time, whereas tidal volume remained constant (see Fig. 4/Table 3).

## Discussion

In preterm infants non-responsive to initial facemask ventilation after birth, there was a rapid increase in FRC after NPT insertion followed by an immediate clinical improvement. This gain in lung volume was not larger than during facemask ventilation, when equally long periods on the respective interface were analysed. Based on our findings, the NPT may be a clinically relevant tool to prevent intubation in selected infants who are non-responsive to facemask ventilation.

In the current study, we used EIT to determine changes in EELI (corresponding to changes in overall lung aeration) before, during and after the insertion of an NPT. We saw that there was a considerable dynamic in the development of ΔEELI, corresponding to the highly adaptive initial phase after birth.^23,24^ In this sample of infants unresponsive to initial facemask ventilation, the largest increase in ΔEELI was noted after insertion of the NPT. Changes in EELI correspond to changes in FRC and thus, this finding may imply an improved lung aeration with the use of an NPT. As the NPT is closer to the glottis, applied pressures may reach the lung more effectively by circumventing the compliant nasopharyngeal space where inflation pressures may be dampened.^25^ Importantly, FRC dropped back to baseline values after removal of the facemask, demonstrating the highly compliant chest wall and illustrating the necessity to maintain the distending pressure during delivery room stabilisation. A potential benefit of the NPT may thus be counteracted by this prior loss in FRC. However, we saw that the steep increase in FRC after insertion of the NPT was persistent to 5 min after insertion where delivered pressure levels were already decreased.

In order to differentiate the noted changes from a mere time effect, we evaluated individual timespans for each patient, which were of the same length during facemask and NPT ventilation. In our small sample, we saw an increase in FRC after insertion of the NPT but this increase was not significantly larger than during facemask ventilation. This is in line with previous clinical studies using the NPT as primary interface after birth.^9,10,26^ The NPT confers the risk of nasopharyngeal trauma and vasovagal reactions, but less than with a laryngoscope during endotracheal intubation.^27,28^ Therefore, it is recommended as rescue interface before impending intubation in some resuscitation guidelines.^29^ Future randomised studies are warranted to prospectively evaluate whether the use of a rescue NPT is superior to facemask ventilation to prevent endotracheal intubation in non-responsive infants.

While there was no clear advantage of the NPT compared with facemask ventilation, single patients seemed to benefit from the NPT while others did not. It remains unclear how to identify preterm infants who may benefit from the use of the NPT. We speculate that apnoeic infants with only limited lung volume recruitment who are clinically unstable with bradycardia and low oxygenation, may benefit more from the use of an NPT. Possibly, the trigeminocardiac reflex leading to apnoea and bradycardia may be induced to a lesser extent when using an NPT but this was never investigated to date.^30^ Future studies should evaluate factors influencing success or failure of using an NPT. We speculate that EIT may be a monitoring tool allowing an individualised approach to respiratory support after birth.

Of note, the development of lung volumes was similar between infants who received SN and the control group. This is unsurprising for three reasons: (1) the original trial did not find any clinically relevant differences between the two groups and thus, we did not expect a large effect in this subgroup of infants in a specific situation, (2) for some infants in the intervention group, nebulization had already finished by the time the NPT was inserted, thus making an additional effect unlikely, and (3) the sample size was very small and this subgroup analysis was not powered to detect marginal differences between the two groups.

An improved aeration increases the infant’s oxygenation, which in turn may improve the respiratory drive.^31–33^ Thus, we speculate that the insertion of the NPT may also be associated with an increased number of spontaneous breaths which may in turn improve oxygenation thereby decreasing the likelihood of apnoea. However, the likelihood of patient-ventilator asynchrony is increased with more spontaneous breaths. Thus, synchronised non-invasive ventilation may be beneficital in the delivery room and is currently investigated.^34^

Previously, provision of NIPPV via NPT compared to facemask was associated with lower tidal volumes and lower oxygen saturation.^10^ In contrast, we saw similar V_T_ and improved oxygenation as well as HR. Most bradycardic infants became normocardic after insertion of the NPT, which indicates a potential clinical benefit despite small effects on FRC. This effect may be mediated by a time effect, by the increased pressures and/or by an increased oxygenation. Since insertion of the NPT happened more than 5 min after birth in most infants, a time effect seems less likely. Conversely, applied inspiratory pressures were increased over time, possibly contributing to cardiorespiratory stability. Larger pressures may be relevant for initial lung aeration, consequently improving oxygenation and strengthening the respiratory drive.^31–33^ Future randomised studies may need to consider standardisation of applied pressures in order to tease out this effect.

There was a positive effect of the NPT on physiological parameters and applied pressures in our study. Handling of the NPT is not trivial as the contralateral nostril and mouth has to be kept closed.^35^ In our study, clinicians could see the respiratory function monitor (RFM) and adjust theira clinical management to optimise ventilation (e.g. closing the nostril, jaw thrust, etc). If physiological parameters are not visible, the NPT might be more difficult to handle compared to a facemask. With a visible RFM, the NPT as rescue interface may improve clinical parameters, and future studies should evaluate the rescue NPT in clinically meaningful studies.

This study has various limitations: First, it is a single-centre study, and other neonatal units with different approaches to neonatal stabilisation might see different results. Second, it is a secondary analysis of a trial on the effect of SN which may have skewed the data. Third, clinical validity of the data is limited due to the small sample of only 15 infants. However, we noted large changes over time, indicating a large effect size. Fourth, due to artefact exclusion, EIT data were restricted to 521 breaths. Fifth, the decision to apply NIPPV via NPT and the adjustment of pressures were the clinician’s choice and therefore, highly individualised. Still, this was the first study to show effects of a rescue NPT during NIPPV, and we demonstrated promising effects on lung volumes and cardiorespiratory stability. Larger prospective trials are needed to evaluate the advantages of an NPT in delivery room stabilisation of preterm infants.

## Conclusions

In this small study, the use of an NPT as rescue interface resulted in a considerable increase in EELI in very preterm infants non-responsive to initial facemask ventilation after birth. While the increase in lung volume was not significantly higher than during facemask ventilation in the same timespan, clinical parameters (SpO_2_ and HR) increased significantly with a clinically relevant magnitude after insertion of the NPT. Our results highlight the importance of individual strategies for respiratory support in preterm infants after birth and indicate that intubation may be prevented in some infants by use of a NPT.

### Supplementary information

Supplementary information

## Data Availability

Deidentified individual participant data will be made available from 3 months to 3 years following publication, in addition to study protocols, the statistical analysis plan, and the informed consent form to researchers who provide a methodologically sound proposal, with approval by an independent review committee (‘learned intermediary’). Data requestors will need to sign a data access or material transfer agreement approved by USZ. Proposals should be submitted to vincent.gaertner@usz.ch to gain access.
