# Supplementary material for: Rescue nasopharyngeal tube for preterm infants non-responsive to initial ventilation after birth
Source: Pediatr Res. 2024 Jan 25;96(1):141–7. doi: 10.1038/s41390-024-03033-6 (PMC11257935; doi:10.1038/s41390-024-03033-6)
Supplement: Supplementary file 1 — Supplementary information [file 41390_2024_3033_MOESM1_ESM.pdf]

Figure S1

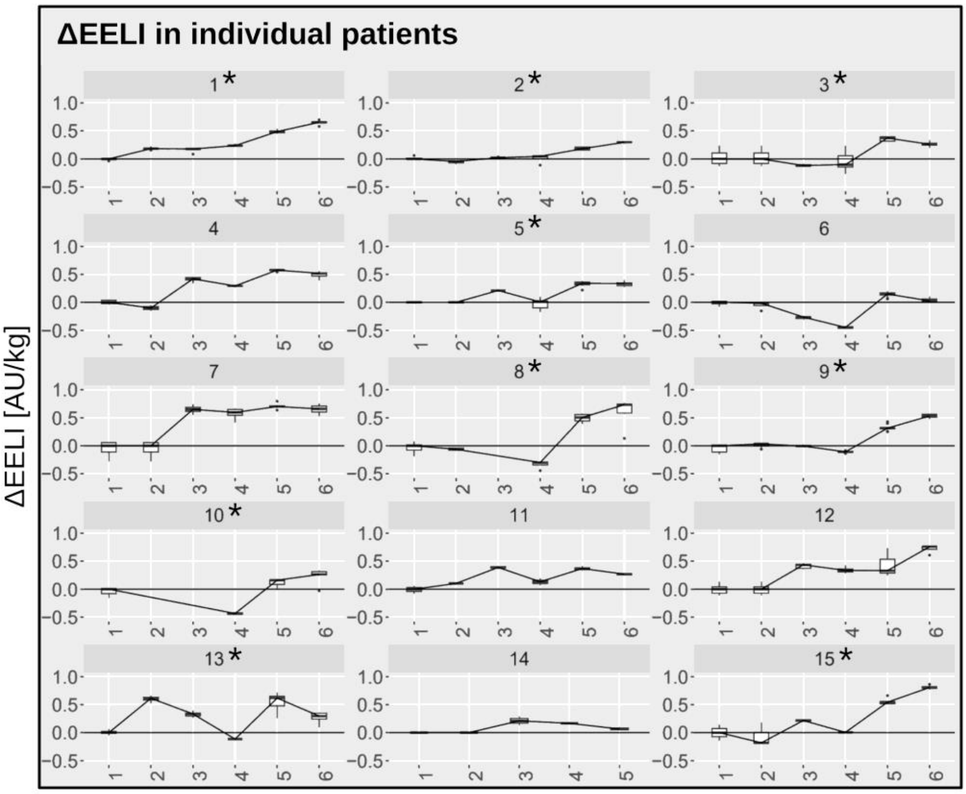

Supplementary Figure S1. Individual development of EELI in all patients.
EELI = changes in end-expiratory lung impedance.

Figure S2

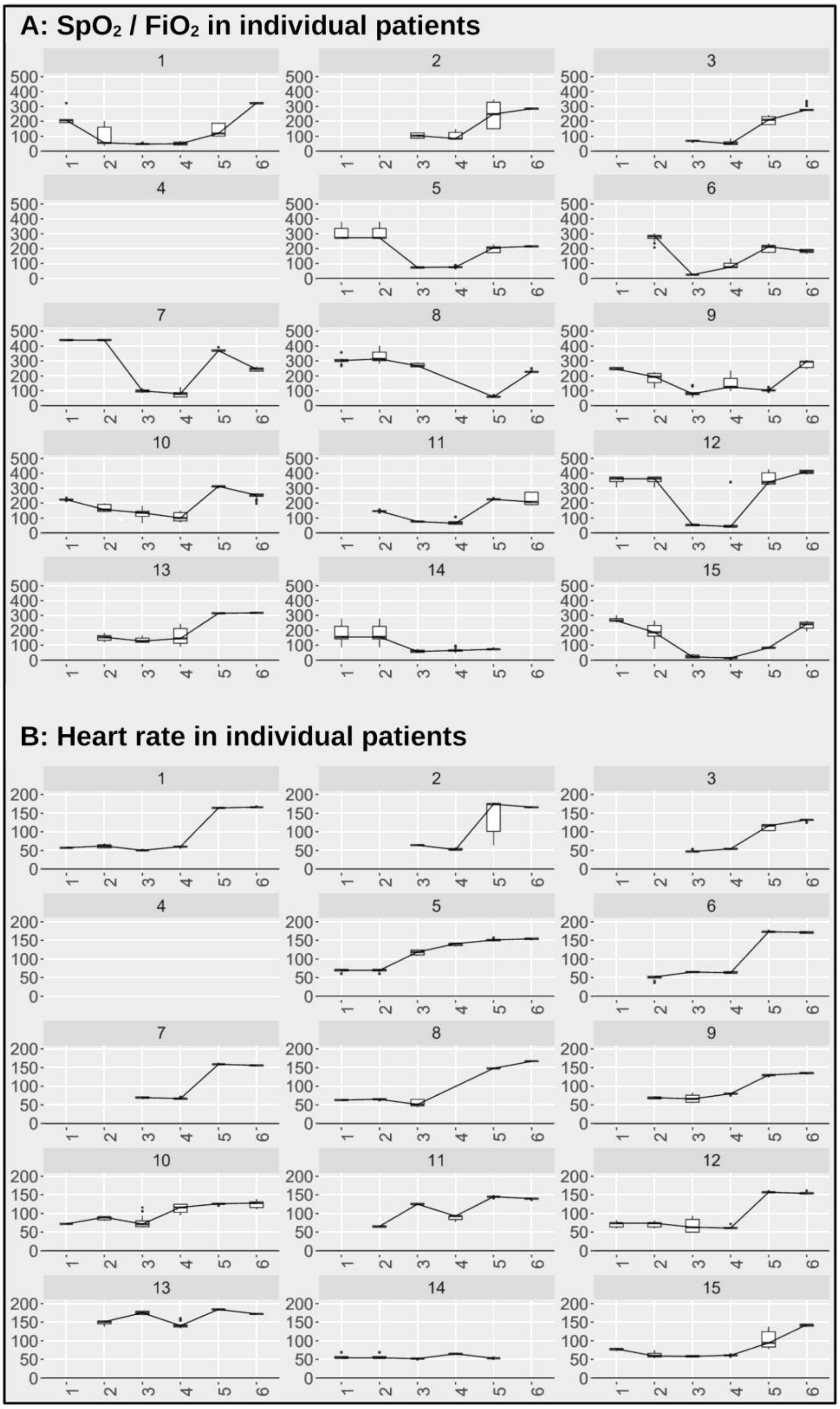

Supplementary Figure S2. Individual development of heart rate and SpO2/FiO2-ratio in all patients.
